# High-efficiency RNA cloning enables accurate quantification of miRNA expression by deep sequencing

**DOI:** 10.1186/gb-2013-14-10-r109

**Published:** 2013-10-07

**Authors:** Zhaojie Zhang, Jerome E Lee, Kent Riemondy, Emily M Anderson, Rui Yi

**Affiliations:** 1 Department of Molecular, Cellular, and Developmental Biology, University of Colorado, Boulder, CO, 80305, USA; 2 Dharmacon Inc., a wholly owned subsidiary of Thermo Fisher Scientific, Inc., Lafayette, CO, 80026 USA

## Abstract

Small RNA cloning and sequencing is uniquely positioned as a genome-wide approach to quantify miRNAs with single-nucleotide resolution. However, significant biases introduced by RNA ligation in current protocols lead to inaccurate miRNA quantification by 1000-fold. Here we report an RNA cloning method that achieves over 95% efficiency for both 5′ and 3′ ligations. It achieves accurate quantification of synthetic miRNAs with less than two-fold deviation from the anticipated value and over a dynamic range of four orders of magnitude. Taken together, this high-efficiency RNA cloning method permits accurate genome-wide miRNA profiling from total RNAs.

## Background

MicroRNAs (miRNAs) are a class of small (approximately 20 to 22 nucleotides), non-coding RNA species that are broadly expressed in almost all eukaryotes. They regulate diverse biological processes by targeting a large number of protein-coding mRNA transcripts [1]. The widespread regulation of mRNAs by miRNAs is supported by the evidence obtained from computational analysis [2,3], transcriptome analysis [4,5] and proteome profiling [6,7]. With recent developments in quantitative mRNA profiling, it is estimated that the median copy number of an mRNA is 17 molecules, and the dynamic range for mRNA copy number is approximately 10^4^ in a single mammalian cell [8,9]. Because miRNAs regulate mRNA expression in a stoichiometric manner [10,11] and a conserved miRNA is predicted to recognize approximately 400 target sites [1,12], such broad variation in mRNA expression suggests miRNAs could differentially regulate highly or lowly expressed mRNAs. Indeed, it was recently demonstrated that miRNAs could function either as a switch or a fine-tuner, depending on the copy number of both miRNAs and their cognate mRNA targets [10]. Adding to the complexity of miRNA-mediated regulation, other RNA species, including competing endogenous mRNAs or circular RNAs, were recently shown to function as a molecular decoy by titrating miRNAs from their functional mRNA targets [13-16]. Collectively, these studies suggest that a highly expressed miRNA is capable of regulating many more mRNA targets and/or repressing mRNA targets more potently than a lowly expressed miRNA within a cell. Therefore, these findings highlight the importance of accurately determining the relative expression levels of different miRNAs in a given cellular context.

Three major platforms, quantitative real-time reverse transcription PCR (qRT-PCR), microarray and small RNA cloning followed by deep sequencing (miRNA-Seq), have been developed for genome-wide miRNA profiling. However, both qRT-PCR and microarray platforms are hybridization-based techniques suitable only for known miRNAs and are unable to provide single-nucleotide resolution for miRNA detection. Furthermore, due to the limitation of probe design for such a short sequence of miRNAs, absolute quantification of individual miRNAs requires extensive optimization and individual internal controls [17]. In contrast, with the rapid development of next-generation sequencing techniques, miRNA-Seq has the potential to provide single-nucleotide resolution of miRNA species, facilitate *de novo* miRNA discovery, and offer nearly unlimited dynamic range for miRNA quantification [18]. The enthusiasm towards miRNA-Seq, however, has been severely dampened by the identification of significant biases introduced during small RNA cDNA library preparation, often more than three orders of magnitude for individual miRNAs [19,20]. Although the cloning biases are likely caused by many factors, the RNA ligation has been identified as one of the major sources [20]. Consistent with this view, recent studies with modifications to the ligation reactions have shown some promise to reduce the cloning bias [21-23]. However, a highly effective method for genome-wide miRNA quantification by deep sequencing has yet to be successfully developed.

In this study, we investigate the highly biased miRNA quantification by current miRNA-Seq methods with 29 representative miRNAs. We demonstrate that inefficient and biased RNA ligation for both 3′ and 5′ adapter ligation is the major contributing factor for the inaccurate quantification. To develop an unbiased RNA cloning protocol, we have performed extensive tests with multiple customized adapters, RNA ligases as well as ligation conditions. As a result, we have established an RNA cloning strategy that allows nearly 100% ligation efficiency for both 3′ and 5′ ligation steps. When applied to a pool of either equimolar or differentially mixed synthetic miRNAs, our method achieves less than two-fold deviation from the expected results for each miRNA. We further demonstrate that the unbiased cloning is applicable to complex RNA mixtures using *Caenorhabditis elegans* total RNA with the synthetic mix spiked in. Finally, we apply our method to biological samples isolated from multiple mouse tissues as well as HeLa cells. Our results provide a quantitative view of miRNA expression for each of these samples. These data also reveal that most cistronic miRNAs that are derived from a single transcript are expressed at similar levels, and that miRNAs repress their target expression in a dose-dependent manner. Taken together, we have established a highly efficient miRNA-Seq method that accurately quantifies individual miRNAs whose expression levels are across four orders of magnitude. Furthermore, the optimized RNA ligation can be readily applied to other RNA ligation-based RNA-Seq methods to provide quantitative insights for RNA transcripts.

## Results and discussion

### Characterization of systematic bias in current miRNA-Seq method

**Figure 1:**
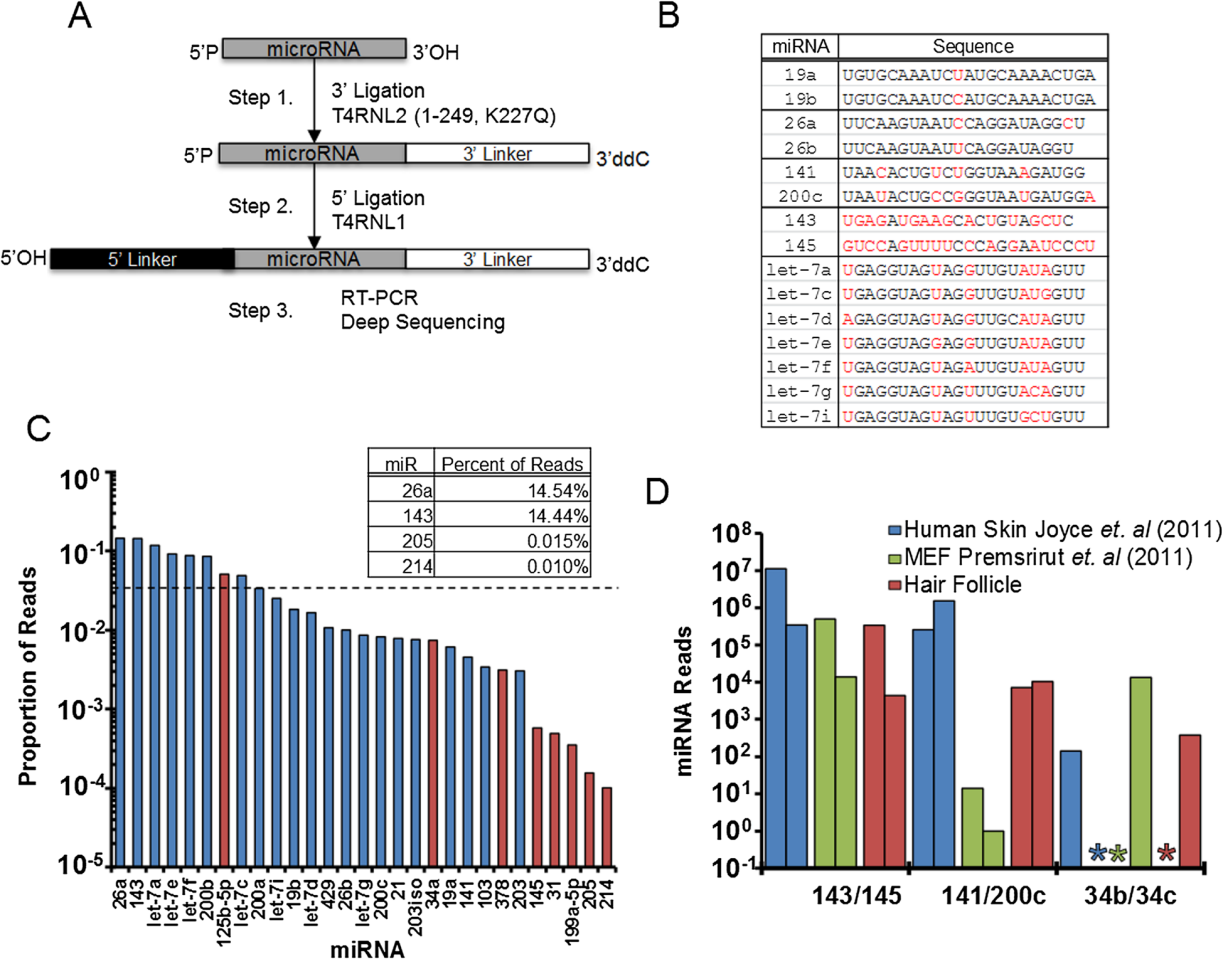
**Current miRNA-Seq method yields inaccurate miRNA quantification. (A)** Schematic of the two-step ligation protocol used to prepare small RNA libraries for deep sequencing. **(B)** Table of representative miRNAs from the 29 synthetic miRNA pool grouped by cluster where sequence differences are in red. **(C)** Representative result of deep sequencing from an equimolar mixture of the 29 synthetic miRNAs. Dashed line indicates expected cloning frequency for all, red bars indicate miRNAs with ≥50% GC content. Note the deviation is more than three orders of magnitude. **(D)** Deep sequencing data from previously published results, and one of our skin samples obtained from mouse hair follicle, grouped by miRNA clusters. Asterisks denote the absence of sequencing reads for a miRNA.

Since the original small RNA cloning technique was developed for *de novo* miRNA discovery, it has played an instrumental role in uncovering the fascinating world of small RNAs that includes miRNAs [24-26], piwi-interacting RNAs (piRNAs) [27,28] and endogenous small-interfering RNAs (siRNAs) [29,30]. To characterize the molecular identity of small RNAs, 3′ and 5′ adapters are sequentially attached to these tiny molecules by RNA ligase (Figure 1A). The ligation steps provide anchor sequences for subsequent reverse transcription and PCR reactions that amplify and convert the pool of small RNA molecules into a double-stranded DNA library (Figure 1A). To investigate the systematic biases and optimize the quantitative performance of the existing method, we synthesized a small but representative pool of 29 miRNAs (Table S1 in Additional file 1). This pool included closely related miRNA family members that differ by one, two or multiple nucleotides at different positions (for example, miR-19, miR-26, let-7 and miR-200 families), miRNAs that are clustered in the same genomic locus but with unrelated sequences (for example, miR-143/145), as well as miRNA isoforms that are derived from the same hairpin but with a single-nucleotide difference (Figure 1B). In addition, we also included in the pool miR-31, one of the least cloned miRNAs from a previous study [20]. When tested with the current protocol, this pool of 29 synthetic miRNAs has recapitulated the >1,000-fold deviation in cloning frequency as reported previously with a large collection of synthetic miRNAs [20] (see below for details). Finally, with the exception of let-7a, which is identical to let-7 in *C. elegans*, 28 out of these 29 miRNAs are also specific to vertebrates. This design allows the synthetic miRNAs to be mixed with total RNAs obtained from *C. elegans* and, in doing so, simulates complex RNA substrates represented in biological samples (see below for details).

We first prepared an equimolar mixture of these 29 synthetic miRNAs and performed miRNA-Seq with the existing method [18,20,31]. Consistent with previous reports [19,20], the cloning frequency of individual miRNAs varied by more than 1,000-fold (Figure 1C). We noted that miR-143, the second highest cloned miRNA in our pool, shows a much higher cloning efficiency (249-fold higher) than miR-145, its clustered, cistronic partner. Remarkably, such bias favoring miR-143 has been observed in many miRNA-Seq experiments using total RNA, including in our results from mouse hair follicles (Figure 1D; Figure S1A in Additional file 1). In many independent studies, miR-143 is consistently reported as one of the most highly cloned miRNAs; in sharp contrast to this, miR-145 often falls outside of the top 50 most cloned miRNAs [32,33] (Figure 1D). However, miR-143 and miR-145 show very similar expression levels when measured by northern blotting [34] or qPCR-based absolute quantification (Figure S1B in Additional file 1). Similar to the results for miR-143/145, when we examined other clustered, cistronic miRNAs from the datasets generated by the current method, we observed significant variation in cloning frequency (Figure 1D). Thus, significant and widespread biases in the current miRNA-Seq method severely compromise the ability to quantify individual miRNAs and hinder the application of miRNA-Seq for identifying highly expressed miRNAs in a given cellular context.

### RNA folding contributes to the systematic bias

**Figure 2:**
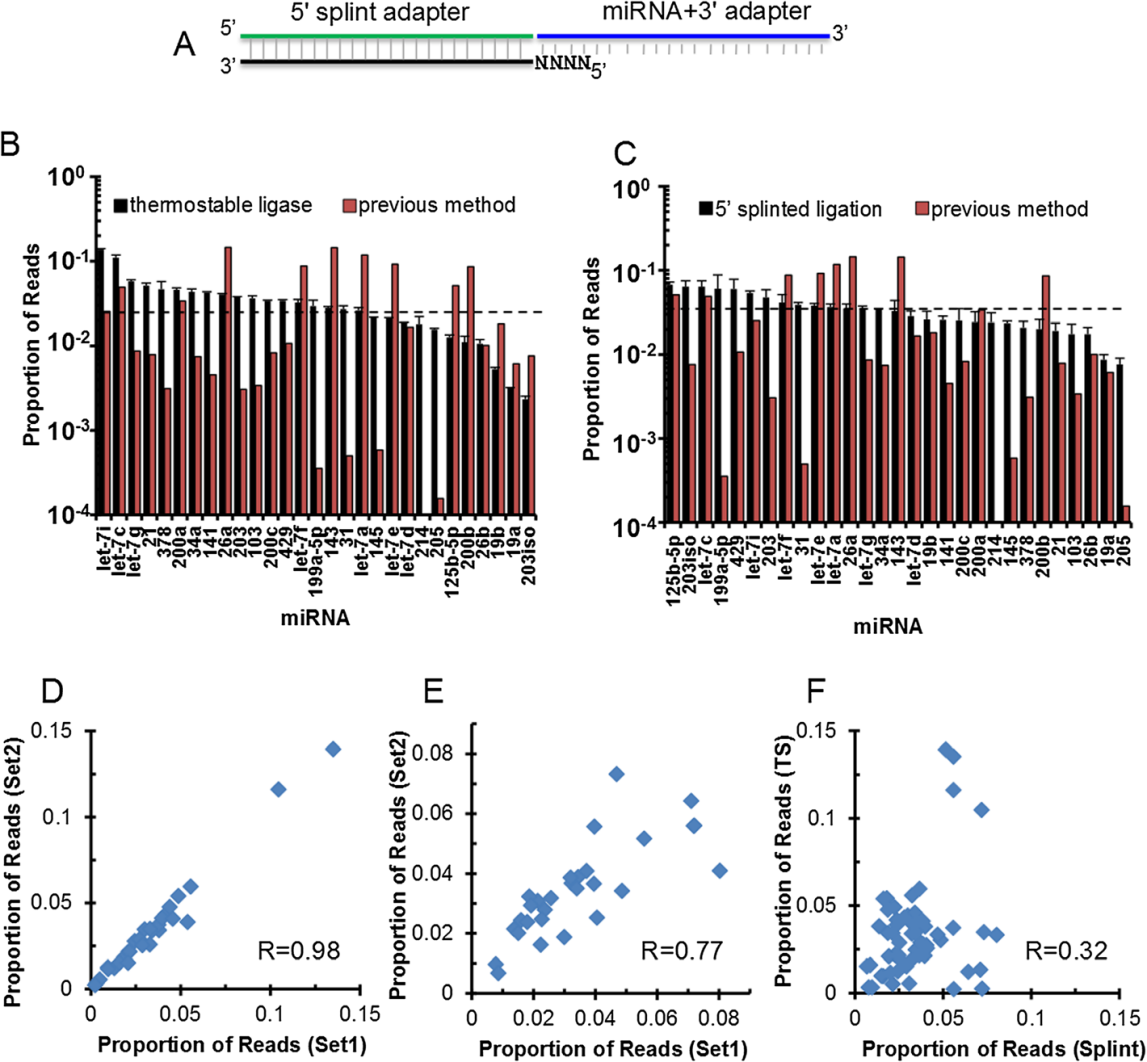
**Improved 5′ ligation reactions reduce cloning bias. (A)** Schematic of the 5′ ligation splint where the DNA splint is shown in black, the 5′ RNA ligation adapter is shown in green, the randomized nucleotides are shown as 'N', and the product of the 3′ ligation (miRNA + 3′ adapter) is shown in blue. **(B)** Representative result of deep sequencing from an equimolar mixture of the 29 synthetic miRNAs using the original cloning method (red bars) and the thermostable RNA ligase for the 5′ ligation (black bars). Dashed line indicates expected cloning frequency. **(C)** Same as in **(B)** except the splint structure shown in (A) is used for the 5′ ligation step. **(D)** Scatter plot amongst replicates of miRNA-Seq done with thermo-stable RNA ligase. **(E)** Same as in **(D)** except splint ligations are being compared. **(F)** Scatter plot of thermo-stable RNA ligation data as a function of splinted ligation. For all plots, error bars represent standard error of the mean.

We noted that miRNAs with >50% GC content were consistently among the poorest cloned ones in our synthetic pool (Figure 1C, red bars; Table S2 in Additional file 1). These results suggest the possibility that stable hybridization between 3′ ligated RNA substrates and the 5′ adapter may inhibit ligation. We then took two approaches to reduce the RNA folding during 5′ ligation. First, we employed a thermostable RNA ligase and performed the 5′ ligation at 60°C instead of room temperature as required for T4 RNA ligase 1. We reasoned that with the elevated temperature we would significantly reduce intermolecular base-pairing. The second approach made use of a splint adapter that is the reverse complement of the full-length 5′ adapter plus a randomized four-nucleotide 5′ overhang at the 3′ end of the 5′ adapter (Figure 2A). This pre-annealed splinted adapter should largely eliminate any intermolecular hybridization between the 5′ adapter and 3′ ligated RNA substrates. Consistent with our hypothesis, both modifications achieved considerable improvement in quantitative representation of individual miRNAs by miRNA-Seq. Compared to >1,000-fold deviation from the anticipated value (each miRNA's cloning frequency is expected to be 3.45% from the equimolar mix) by the current method (Figure 1C), the deviation was reduced to approximately 14.7-fold by the thermostable ligase (Figure 2B) and to approximately 4.5-fold by the splint adapter (Figure 2C). When we repeated the experiments under identical conditions for each modification, we obtained reproducible results (r = 0.98 and 0.77, respectively; Figure 2D,E). However, the correlation coefficient between these two approaches is low (r = 0.32; Figure 2F). This suggests that different ligation reactions have variable efficiency for individual miRNAs.

### High-efficiency RNA ligation

**Figure 3:**
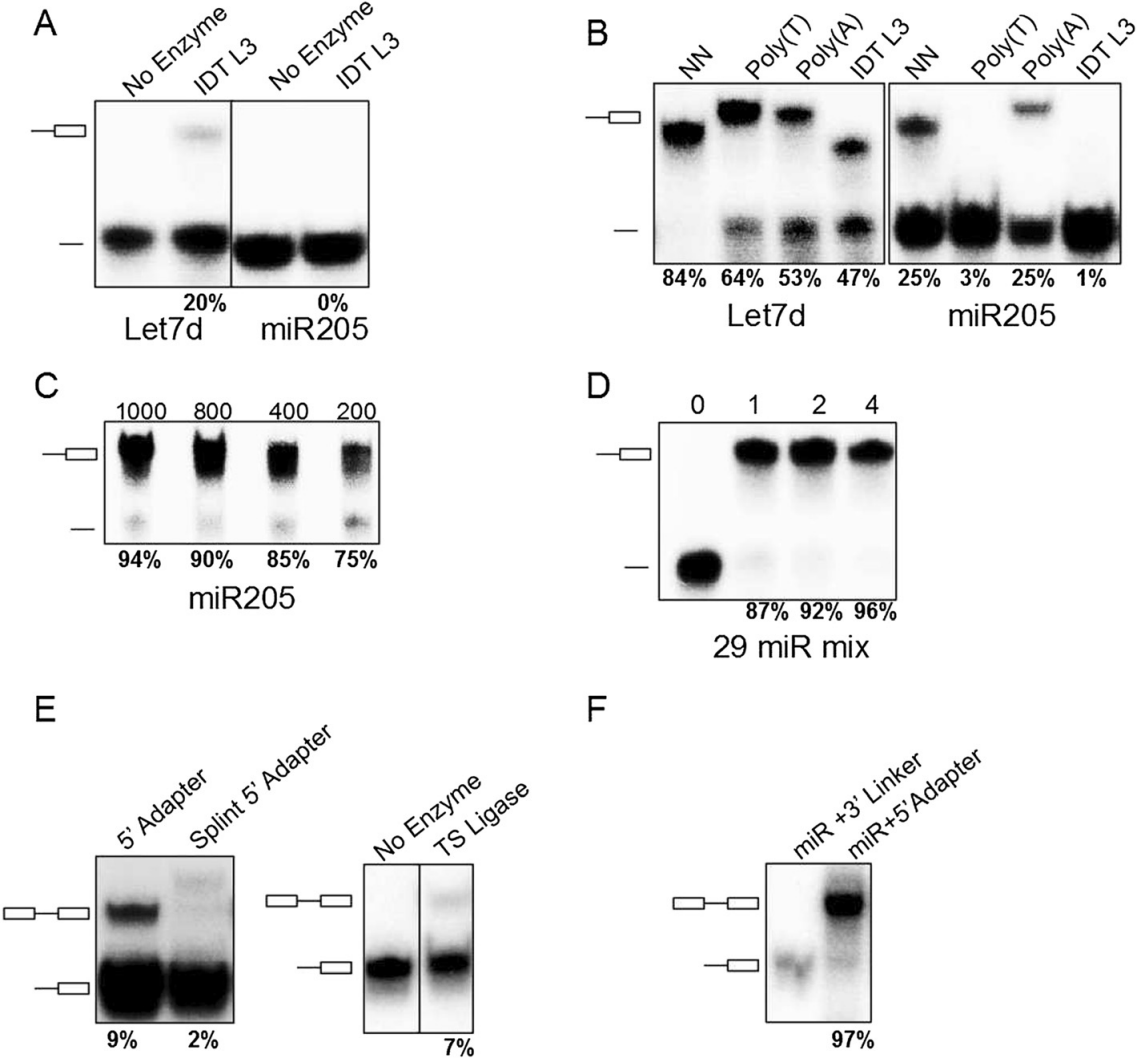
**Optimized RNA ligation conditions achieve high efficiency for both 3′ and 5′ ligation steps. (A)** 3′ ligation efficiencies were determined by resolving ligation reactions with radiolabeled synthetic miRNA substrates and Linker-3 (Integrated DNA Technologies) on 15% Urea-PAGE. A dash indicates free miRNA; a dash with a box  indicates 3′ ligation product. The numbers at the bottom indicate the percentage of ligated species relative to the total. **(B)** 3′ ligation efficiencies were tested as in **(A)** with varying 3′ linkers and 10% w/v PEG8000. **(C)** 3′ ligation efficiency was enhanced by increasing the amount of NN linker used. The numbers at the top indicate fold molar excess of linker to substrate RNA. **(D)** A mixture of 29 synthetic miRNAs were radiolabeled and subjected to enhanced 3′ ligation conditions in the presence of 0.5 μg of total RNA obtained from mouse skin. The numbers at top indicate time in hours the reaction was carried out. **(E)** 5′ ligation efficiencies were assessed using gel-purified, radiolabeled, 3′ ligated, equimolar 29 synthetic miRNA mixtures with T4 RNL1 (left panel) at room temperature or with thermostable (TS) RNA ligase at 60°C (right panel) for 2 hours. The numbers at the bottom indicate the ligation efficiency, and a dash between two boxes indicates 5′ ligation product. **(F)** Optimized 5′ ligation reaction achieves 96% ligation efficiency.

We then examined RNA ligation efficiency for both 3′ and 5′ ligation steps with different ligases and RNA substrates. Because let-7d was routinely found in our miR-Seq dataset at expected or greater than expected levels and miR-205 was often under-represented, we selected these two miRNAs for the initial test. We first 5′ end-labeled let-7d and miR-205 with ^32^P and examined 3′ ligation efficiency with a previously published 3′ linker [24]. We observed low ligation efficiency for both substrates. Whereas let-7d showed modest levels of ligation, miR-205 was a particularly poor substrate in the reaction (Figure 3A). This result prompted us to include polyethylene glycol in subsequent reactions and to design several additional 3′ adapters, as previous reports indicate these factors influence ligation preference [21,35]. We synthesized DNA oligonucleotides beginning with T_10_, A_10_, and NN randomized adapters followed by Illumina 3′ adapter sequences (see Table S3 in Additional file 1 for details) and converted them into pre-adenylated adapters with a recently established reaction (Figure S2 in Additional file 1) [22]. Interestingly, miR-205 and let-7d showed strong preferences for 5′-A and 5′-T, respectively (Figure 3B). Importantly, both miR-205 and let-7d ligated most efficiently to the 5′-NN randomized adapter (Figure 3B). These observations suggest that different miRNAs exhibit different preferences for the 5′ nucleotide(s) of the 3′ adapter, and that the randomized adapter has the best potential to be ligated to diverse RNA substrates with high efficiency. Furthermore, we reasoned if ligation efficiency can be enhanced to near 100% for all miRNAs, the bias introduced by RNA ligase in the miRNA-Seq method could be largely eliminated. Previous studies of individual RNA ligation reactions suggest that a high concentration of polyethylene glycol (PEG)8000 improves ligation efficiency [35], and that RNA ligation efficiency is correlated with the concentration of RNA ligase [36], and that dimethylsulfoxide (DMSO) stimulates ligation to highly structured tRNAs [37]. We therefore tested all of these conditions and determined that with 10% PEG8000 and gradually increased 3′ adapter concentration we could approach 100% ligation efficiency even for miR-205, a particularly poor substrate (Figure 3C). To test whether our optimized condition is suitable for a broad range of substrates, we labeled a mix of all 29 synthetic miRNAs and performed the 3′ ligation. As shown in Figure 3D, all RNA substrates were effectively ligated by a 2-hour ligation reaction.

Having established an effective ligation method for the 3′ ligation, we re-visited the 5′ ligation. Similar to the 3′ ligation, the 5′ ligation efficiency in the current method was also very low. We observed 9% efficiency collectively for the mixed synthetic miRNAs (Figure 3E). In addition, the ligation efficiency for the thermostable ligase and the 5′ splint adapter was also very low at 7% and 2%, respectively (Figure 3E). These results prompted us to optimize the 5′ ligation efficiency. We took a similar approach based on our success in the 3′ ligation. This included: 1) the use of 3′ end NN randomized adapter; 2) further increasing the PEG8000 concentration to 20% in the reaction buffer; and 3) elevating the reaction temperature to 37°C. As shown in Figure 3F, these modifications optimized the 5′ ligation to approximately 97% efficiency. Taken together, these results demonstrate that we have established a highly efficient method for attaching both 3′ and 5′ adapters to miRNA substrates.

### High-efficiency RNA ligation allows quantitative measurement of synthetic miRNAs by miRNA-Seq

**Figure 4:**
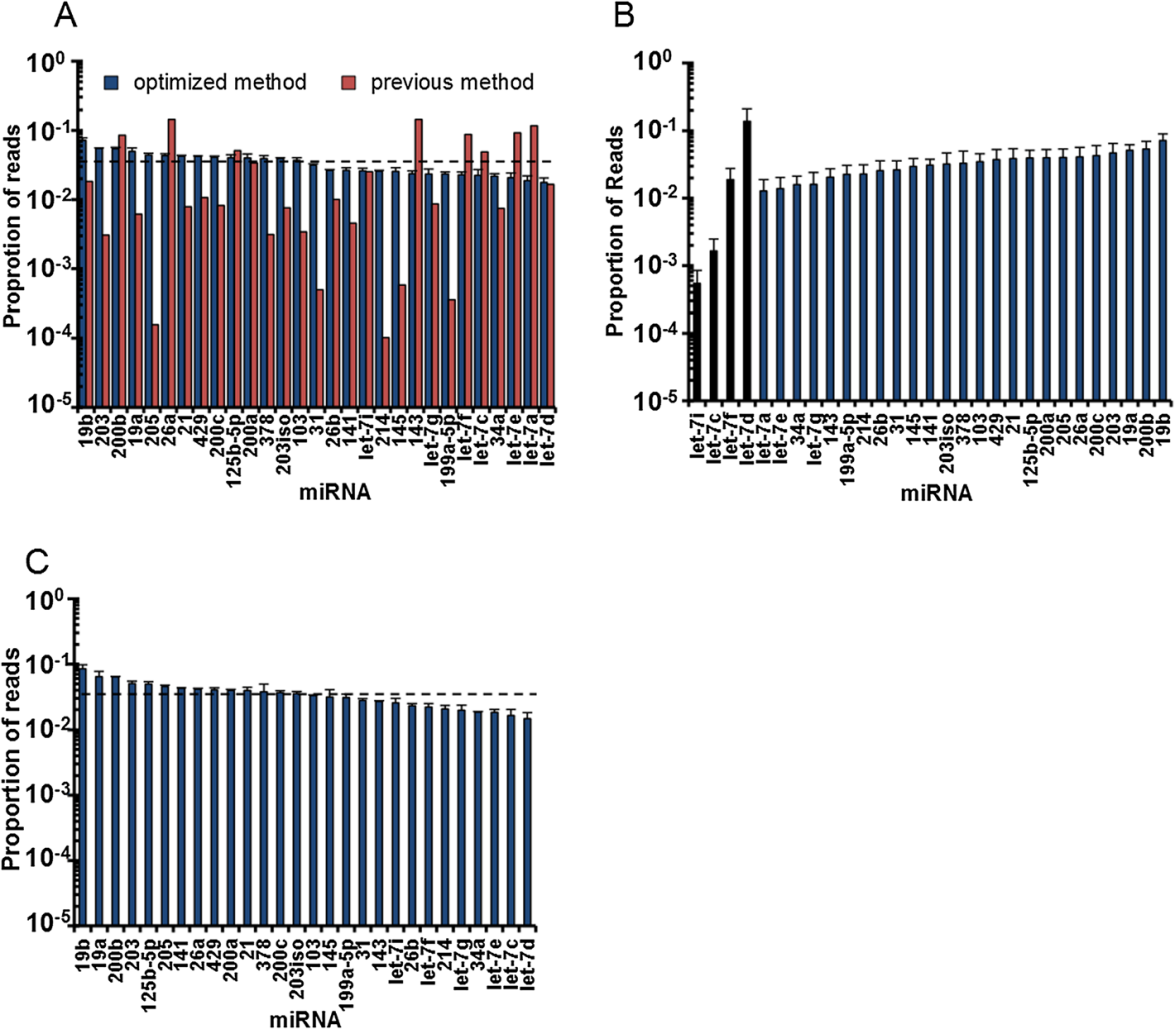
**High efficiency ligations capture synthetic miRNAs with high fidelity. (A)** Deep sequencing data of an equimolar mixture of 29 synthetic miRNAs from high efficiency ligation reaction (blue bars) and previous method (red bars); the dashed line indicates expected result. For all high efficiency ligations, plotted is the average of at least two independent experiments where error bars represent the standard error of the mean. **(B)** As in **(A)** except the mix is composed of an equimolar mixture of 25 synthetic miRNAs (blue bars) and differential mixture of 4 let-7 miRNAs (black bars) where the ratio was let-7i:let-7c:let7f:let-7d = 0.01:0.1:1:10. **(C)** As in **(A)** except the synthetic mixture was combined with 0.5 μg *C. elegans* total RNA prior to ligations.

We next applied our optimized ligation method to miRNA-Seq. We first tested with the equimolar mix of 29 synthetic miRNAs. As shown in Figure 4A, our method drastically improved the quantitative performance of miRNA-Seq. In sharp contrast to the >1,000-fold deviation obtained by the current method, each miRNA was cloned within 1.8-fold deviation by our optimized miRNA-Seq. The reproducibility of our method was high (r > 0.9) even when we used different amounts of input RNAs (Figure S3 in Additional file 1). We further tested the dynamic range of our optimized method by mixing four individual let-7 members at concentrations across 4 orders of magnitude together with 25 other synthetic miRNAs. We observed a cloning ratio of 8.0:1.0:0.1:0.03 for the four let-7 members, compared with the anticipated ratio of 10:1.0:0.1:0.01 (Figure 4B). This further validated that our method is quantitative over a broad dynamic range (approximately 10^4^). Finally, to examine the performance of our approach with biological samples, we made use of a more complex RNA mixture where *C. elegans* total RNA was spiked with 28 vertebrate-specifi miRNAs. When we examined the cloning frequency of these 28 miRNAs, they were indistinguishable from the results obtained from synthetic miRNA-only reactions (Figure 4C). This result indicates that our optimized miRNA-Seq is applicable to total RNA samples. Collectively, these results demonstrate that our optimized miRNA-Seq method has achieved a highly quantitative miRNA measurement with a broad dynamic range.

### Quantitative miRNA-Seq provides insights into miRNA expression in biological samples

**Figure 5:**
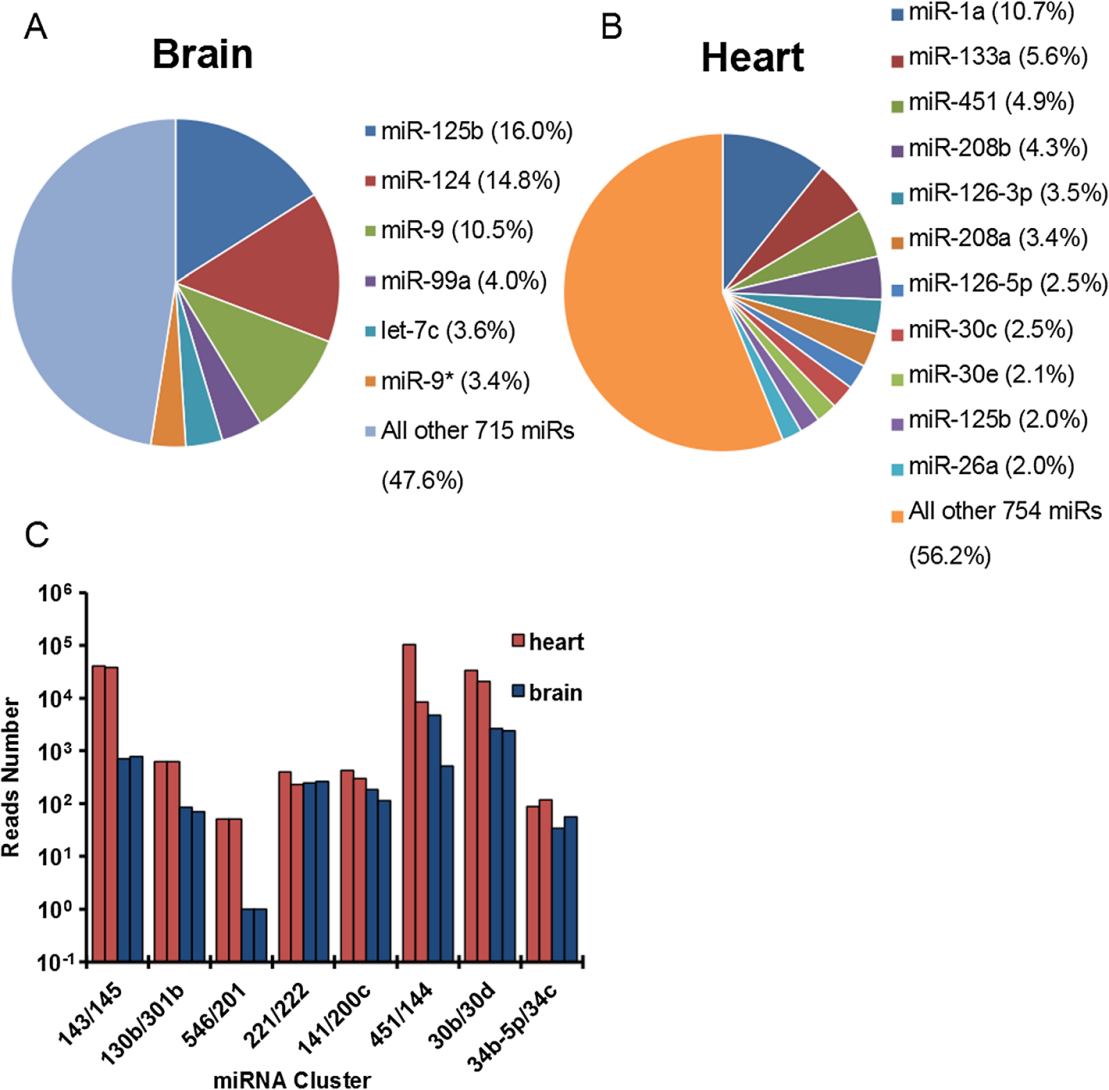
**Optimized miRNA-Seq faithfully captures miRNAs from biological samples. (A)** Deep sequencing data of mapped miRNA reads cloned from total RNA isolated from mouse brain and **(B)** heart. The most cloned miRNAs are shown with the cloning frequencies. **(C)** Deep sequencing data of miRNA reads for cistronic miRNAs obtained from mouse heart and brain. Note the highly similar representation of each cistronic miRNA by read numbers across four orders of magnitude, except for the miR-451/144 cluster.

We then applied our quantitative miRNA-Seq to biological samples obtained from neonatal mouse (postnatal day 7.5) heart and brain in which many individual miRNAs have been functionally characterized. Consistent with previous individual studies, we identified miR-125-5p, miR-124 and miR-9/9* as the most abundantly expressed miRNAs in the brain [38-42] and miR-1, miR-133a, miR-451, miR-208 and miR-126 as the most abundantly expressed miRNAs in the heart (Figure 5A,B) [43-46]. Interestingly, for both brain and heart, the total miRNA pool is dominated by a small number (approximately 10) of highly expressed miRNAs, although more than 700 miRNAs were detectable in both samples (Figure 5A,B). To further examine the quantitative performance of our method in these biological samples, we analyzed the cloning frequency of several clustered, cistronic miRNAs in both samples. We obtained very similar cloning frequency for many of these clustered miRNAs across a broad range of cloning frequencies (Figure 5C). For example, two highly expressed miRNAs in the heart but not in the brain, miR-143 and miR-145, showed very similar read numbers within each sample despite their expression levels differing by 50-fold amongst the two samples (Figure 5C). As an example of lowly expressed miRNAs, miR-546 and miR-201 were both cloned 51 times in the heart and yet only one time in the brain (Figure 5C). The ability to clone these clustered miRNAs very similarly in each biological sample indicates that our method quantitatively represents miRNAs likely across four orders of magnitude in biological samples, similar to the benchmark with synthetic miRNAs (compared with Figure 4B). We also noted that among all of the clustered miRNAs examined, miR-451 and miR-144 consistently showed approximately 10-fold differential expression in both heart and brain (Figure 5C). This is likely explained by the fact that miR-451’s biogenesis is uniquely Ago2-dependent and Dicer-independent [47-49], unlike all other miRNAs known to date, including miR-144. Thus, such differential expression captured by our method is likely reflective of the differential processing efficiency between different miRNA biogenesis pathways.

**Figure 6:**
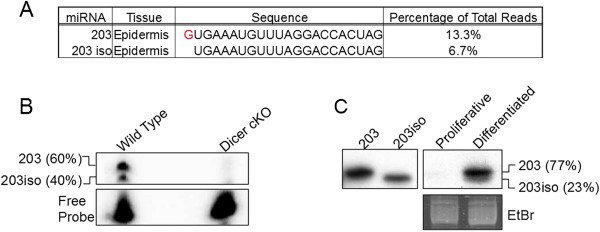
**Identification and quantification of the major miR-203 isoforms in skin by deep sequencing. (A)** Table showing miR-203 with differential 5′ ends (shown in red). **(B)** Primer extension for miR-203 using RNA isolated from mouse total epidermis where upper band corresponds to miR-203 and lower corresponds to miR203iso. Dicer conditional knock out skin sample was used as a control. **(C)** Northern blot probed for miR-203. Left panel shows synthetic miRNA controls, and right panel shows mouse keratinocyte cultures where miR-203 was lowly expressed under the proliferative condition and highly induced in the differentiated condition. Staining of tRNAs by ethidium bromide (EtBr) is shown as the loading control.

Quantitative miRNA-Seq also provides the opportunity to accurately quantify miRNA isoforms differing by only one nucleotide. Although most miRNA isoforms vary at the 3′ end, these sequence changes are thought to have little impact on targeting as the 5′ seed region is the primary determinant for target recognition [1]. However, miRNA isoforms that differ at the 5′ end would cause a shift in seed sequence, likely altering their target recognition. In a recent study, we identified *in vivo* targets of miR-203, a highly expressed miRNA in the skin. We found that down-regulated mRNAs upon miR-203 induction showed enrichment for not only the 2-8 seed matches in their 3′ UTRs, but at positions 3-9 as well [50]. Although the 2-8 seed match is expected, the 3-9 seed match on the mRNA indicates a shifted seed region. We then examined the cloning results for miR-203 in the skin. Interestingly, we found a major miR-203 isoform that was trimmed by one nucleotide from the 5′ end (Figure 6A). Importantly, this isoform was cloned at approximately 50% of the frequency of the canonical miR-203 (6.7% compared to 13.3% total cloning frequency). The apparent 3-9 seed match we observed previously was likely the result of targeting of the shortened isoform at position 2-8. To validate the expression of these two miR-203 isoforms, we performed 5′ end primer extension analysis that allows specific detection and quantification of each isoform. As expected, the canonical form was indeed expressed at approximately two-fold higher than the shortened isoform (Figure 6B). As a control, we did not detect any signal in a Dicer knockout sample where all miRNAs, including miR-203, are depleted [31]. To further confirm the identity of these two isoforms, we performed northern blotting on RNA isolated from mouse total epidermis and again observed two differentially expressed miR-203 isoforms that differ by a single nucleotide (Figure 6C). Taken together, these results further illustrate the application of our quantitative miRNA-Seq in the identification of functionally important miRNA species with single-nucleotide resolution.

### Differentially expressed miRNAs repress their targets in a dose-dependent manner

**Figure 7:**
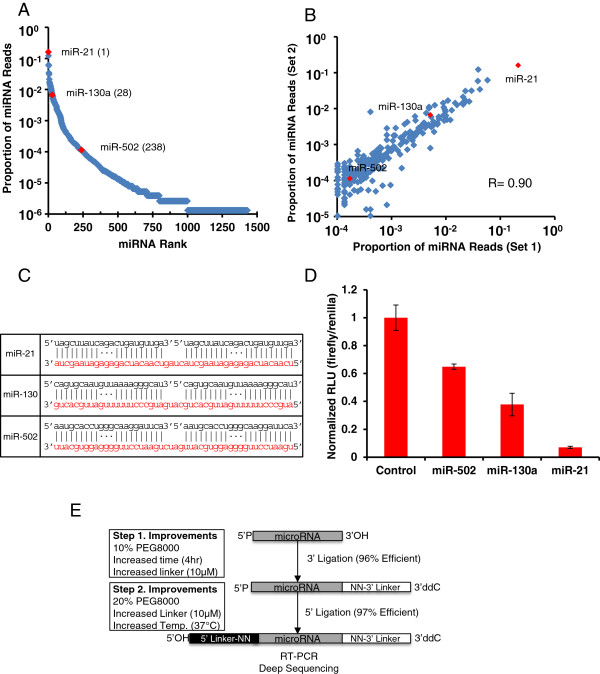
**The potency of miRNA-mediated repression correlates with expression levels. (A)** miRNA-Seq results from 1 μg of HeLa total RNA. Red dots indicate miRNAs of interest with their rank by cloning frequency shown in parentheses. **(B)** Scatter plot of the top 250 miRNAs from two HeLa miRNA-Seq experiments; miRNAs of interest are in red. **(C)** Schematic of the luciferase targeting constructs for miR-21, miR-130 and miR-502. The red sequences are the tandem target sites located in the 3′ UTR of the luciferase mRNA, and the black sequences are the miRNAs. **(D)** Ratios of firefly to renilla luciferase activities in HeLa cultures transfected with miRNA-reporter constructs. Shown is a representative result from three independent experiments. Error bars are the standard deviation from a single experiment where transfections were done in quadruplicate for each construct. **(E)** Schematic depicting the workflow for the optimized miRNA-Seq approach to profiling miRNAs.

Having demonstrated the quantitative capacity of our optimized miRNA-Seq, we asked whether we can leverage the information of relative expression level of individual miRNAs to determine their functional potency in a cellular context. Because miRNAs function in a dose-dependent manner [10], we reasoned that a highly expressed miRNA would exert a stronger degree of repression on its targets than lowly expressed ones. To test this hypothesis, we performed miRNA-Seq with HeLa cell total RNAs and determined miR-21 as the most highly expressed miRNA in these cells (Figure 7A). In addition, independent experiments with these biological samples were also highly reproducible (Figure 7B). To determine whether highly expressed miRNAs are indeed more potent in repressing their targets, we generated a series of heterologous luciferase reporters for three miRNAs expressed at different levels in HeLa cells (Figure 7A). Each reporter contained 2× targeting sites for each miRNA with a central bulge located between 10 to 12 nucleotides (Figure 7C). As expected, miR-21, the most highly expressed miRNA, showed strongest repression; miR-130a, a modestly expressed miRNA, showed intermediate repression; whereas miR-502, a lowly expressed miRNA, showed weak repression of the luciferase activity (Figure 7D). This result supports the functional significance of highly expressed miRNAs in a specific cellular context and that our quantitative miRNA-Seq allows rapid identification of these miRNAs in a high-throughput manner.

## Conclusions

In this study, we have developed a highly efficient miRNA-Seq method to quantify individual miRNAs within a biological sample (Figure 7E). Although the RNA-ligase-introduced bias has been widely recognized [19-22], a systematic solution generally applicable for a broad spectrum of small RNA substrates has not previously been developed. Such a bias, often leading to more than 1,000-fold deviation, has significantly hindered the utilization of miRNA-Seq for quantitative miRNA profiling in a high-throughput manner. Although in some studies the sequencing depth has approached billions of reads [33], the power of accurate quantification of miRNAs based on the cloning frequency remains poor [20]. Because intermolecular RNA hybridization contributes to bias, the application of a thermostable RNA ligase or a 5′ splint adapter significantly reduces such bias. However, the ligation efficiency is low and the improvement by each strategy appears to vary for different RNA substrates. By examining individual steps during the small RNA cDNA library preparation, we have defined optimal conditions to achieve high ligation efficiency for both 3′ and 5′ ligation steps. We determined that the nucleotide composition of the linkers, the concentration of linkers, molecular crowding through the use of PEG8000, and ligation temperature are the primary determinants for highly efficient RNA ligation. We demonstrated with a wide spectrum of small RNA substrates that the ligation efficiency can approach 100%, a drastic improvement compared with the existing method. As a result, the high-efficiency RNA cloning leads to quantitative measurement of miRNAs by miRNA-Seq. To achieve such quantitative results for a pool of approximately 700 miRNAs for a single sample, we usually need approximately 4 to 5 million reads. With the data output routinely over 200 million reads from a single lane of a sequencer, in combination with the multiplexing capacity of the Illumina Hi-Seq platform, our miRNA-Seq method provides an extremely cost-effective and accurate approach for large scale miRNA analyses from numerous samples.

The ability to measure relative expression levels of different miRNAs within a single sample also offers an important approach to identify functionally significant miRNAs. Conventional gene expression analysis by qRT-PCR and microarray usually characterizes differential expression levels of individual miRNAs and identifies miRNAs with the largest fold changes from two or more samples. With the detection sensitivity of qRT-PCR approaching single molecules, some very lowly expressed miRNAs could be identified as highly differentially expressed, though their biological importance may be limited due to low copy number. Since miRNAs function by guiding the RNA-induced silencing complex to their cognate mRNAs and sequestering the mRNAs from productive translation [51], the ratio between miRNAs and mRNA targets is a key factor in determining the potency of a miRNA [10]. In support of this, we demonstrate that the most highly expressed miRNA in HeLa cells, miR-21, has the highest capacity to repress the reporter expression, and the intermediately and lowly expressed ones (for example, miR-130a and miR-502) show much weaker repression. Thus, functionally important miRNAs could be identified as the most highly expressed miRNAs within a cellular context without the knowledge of differential expression patterns. Interestingly, in our analysis of miRNAs in both brain and heart, we observed that a small number (approximately 10) of miRNAs count for a major portion of the miRNA pool. This raises a possibility that the function of the miRNA pathway could be mediated by a handful of highly expressed miRNAs in each cell population.

Finally, our approach to enhance RNA ligation efficiency offers a general guide to improve all RNA ligation-based RNA-Seq methods. The enhanced ligation not only reduces the required amount of RNA input but also, more importantly, improves the representation of different RNA species/fragments in the sequencing libraries. These improvements will allow quantitative analysis of gene expression by fully harnessing the power of deep sequencing.

## Materials and methods

### Linker adenylation

DNA oligonucleotides to serve as 3′ linkers for miRNA cloning were ordered from Integrated DNA Technologies Coralville, Iowa, USA(see Table S3 in Additional file 1 for sequences). All oligonucleotides were ordered with a 5′ phosphate group; those used in sequencing experiments also included a 3′ dideoxycytosine. Prior to ligation, 3′ linkers were adenylated at the 5′ end using the MTH adenylation kit (New England Biolabs #E2610L Ipswich, Massachusetts, USA). Oligonucleotides were phenol/chloroform extracted, precipitated with 2.5 volumes of ethanol, and quantified by A_260_. Adenlyation was confirmed by 18% PAGE (Figure S2 in Additional file 1).

### 3′ ligation reactions

Five picomoles of indicated synthetic miRNA were 5′ end labeled using T4 polynucleotide kinase (Fermentas Hanover, Maryland, USA) with 25 μCi of γ-(^32^P) ATP (3,000 Ci/mmol). Labeled RNA was phenol/chloroform extracted, ethanol precipitated, and resuspended in distilled H_2_O. Initial reactions (Figure 3A) were composed of 25 femtomoles of labeled RNA, 1 picomole of 5′ adenylated DNA cloning linker, 50 mM Tris-HCl pH 7.5, 10 mM MgCl_2_, 1 mM dithiothreitol, 20 units truncated T4 RNA Ligase 2 (residues 1 to 249, K227Q point mutant) (New England Biolabs M0242S Waltham, Massachusetts, USA), and 20 units RNase Inhibitor (Invitrogen N2611 Carlsbad, California, USA). Ligations were permitted to proceed for 90 minutes at room temperature. Reactions were stopped by the addition of an equal volume of 2X loading dye (7 M urea, 1× TBE, 0.01% bromophenol blue and 0.005% xylene cyanol), briefly heated to 95°C, and snap cooled on ice. RNA species were resolved on 15% acrylamide gels containing 7 M urea, exposed to a phosphorimager screen, and visualized by scanning on a Storm Imager (GE Healthcare Piscataway, New Jersey, USA). Subsequent, high efficiency reactions were identical with the following modifications; a final concentration of 10% (w/v) PEG molecular weight 8000 Da (PEG 8000) was included. Reaction times were increased from 90 minutes to 4 hours, and 3′ linker concentration was increased to 10 μM.

### 5′ ligation reactions

Synthetic 5′ ^32^P labeled RNA was subject to 3′ ligation and resolved on 15% PAGE-urea gels as described previously. The ligated product was excised and eluted and served as substrate for 5′ ligation tests. Initial 5′ ligation reactions were composed of the following: 50 mM Tris-HCl pH 7.5, 10 mM MgCl_2_, 1 mM dithiothreitol, 1 mM ATP, 20% (v/v) DMSO, 10 units T4 RNA Ligase 1 (New England Biolabs M0204S), 20 units RNase Inhibitor (Invitrogen N2611), 5 μM 5′ RNA cloning linker, and 25 femtomoles of eluted substrate RNA. Reactions were carried out at room temperature for 2 hours. Subsequent high efficiency ligations were identical with the following modifications: no DMSO, 20% (w/v) PEG8000, 5′ RNA cloning linker increased to 10 μM, and temperature increased to 37°C. Reactions were stopped, resolved, and visualized as described above. For the thermostable ligation, a thermostable RNA ligase (Epicentre TRL8101K Madison, Wisconsin, USA) was used following the manufacture’s instruction.

### Synthetic RNA library preparation

RNA oligoribonucleotides were produced by Thermo Fisher Scientific (Lafayette, Colorado, USA)(for complete list and sequences see Table S1 in Additional file 1). Oligos were resuspended in distilled H_2_O and mixed at an equal volume to generate an equimolar mixture of the 29 synthetic RNAs.

### Small RNA cloning

RNA samples (5 to 1.25 picomoles for synthetic samples and 1 to 4 μg of total RNA for biological samples) were subject to high efficiency 3′ ligation as described above. Products were resolved on 15% PAGE-urea gels and stained with SybrGold (Molecular Probes S-11494 Carlsbad, California, USA). The region corresponding to ligated miRNAs (46 nucleotides) was excised, gel slices were thoroughly minced, and eluted in HSCB (400 mM NaCl, 25 mM Tris-HCl pH 7.5, 0.1% SDS) overnight at 4°C. Nucleic acids were precipitated in the presence of glycogen carrier via 2.5 volumes ethanol and 0.1 volumes sodium acetate. Pellets were washed in 70% ethanol, and resuspended in the 5′ ligation mix without enzyme. Samples were briefly heated to 70°C, snap chilled on ice, and enzyme added. Following ligation reactions cDNA was prepared using Superscript III RT (Invitrogen 18080-085) according to the manufacturer′s recommendation using 3′ linker-specific RT primer. Subsequent cDNA libraries served as templates for PCR amplification; amplicons were resolved on 8% native acrylamide gels. Bands of the correct molecular weight were isolated and used for high-throughput sequencing on the Illumina HiSeq2000.

### Read mapping

A customized miRNA reference database was built with mature miRNA sequences obtained from miRBase [52]. Sequencing reads were mapped to the customized database with BLAST [53].

### Primer extension

A 17-bp DNA primer reverse complementary with nucleotides 6 to 22 of the canonical sequences of miR-203 was used in the primer extension experiment to detect the 5′ length variants of miR-203. Each isoform was quantified using Image J software where the signal from the two bands was summed and each presented as a proportion of the total signal. The assay was performed following the manufacture’s instructions (Promega #E3030 Madison, Wisconsin, USA).

### Northern blotting

Total RNA (10 μg) was electrophoresed through 20% denaturing urea-PAGE gels and electrophoretically transferred to GeneScreen Plus® Nylon Membranes (Perkin-Elmer, Inc. Waltham, Massachusetts, USA). Radiolabeled oligonucleotides complementary to mature miR-203 sequence (miRbase accession MIMAT0000236) were hybridized to the membranes in ExpressHyb Hybridization Solution™ (Clontech Laboratories, Inc. Mountain View, California, USA). Synthetic miR-203 and miR-203iso were loaded as size standards. Bands were quantified as described in the primer extension experiment.

### Luciferase assay

HeLa cell cultures were propagated according to ATCC guidelines. Cells were plated in 24-well trays at a density of 0.1 × 10^6^ cells per well. Sixteen hours post-plating cells were transfected with LT1 (Mirrus Bio Madison, Wisconsin, USA) according to the manufacturer’s instructions with 20 ng of the following constructs: pGL3 control, pGL3-miR502, pGL3-miR130a, and pGL3-miR21 along with 2 ng of Renilla (pRL-CMV), and 378 ng of 'filler' DNA (pLL3.7). Twenty-four hours post-transfection cells were assayed for luciferase activity using the Promega Dual Luciferase Assay Kit according to the manufacturer’s instructions. Data are the ratio of firefly to renilla luciferase activity, normalized to the control construct. Shown is a representative result from an experiment done in triplicate; error bars represent standard deviations.

### Data access

All miRNA-Seq data have been deposited to the National Center for Biotechnology Information Gene Expression Omnibus with accession number GSE47858.

## Abbreviations

DMSO: Dimethylsulfoxide; miRNA: Micro RNA; miRNA-Seq: Small RNA cloning followed by deep sequencing; PEG: Polyethylene glycol; qRT-PCR: Quantitative reverse-transcription polymerase chain reaction; UTR: Untranslated region.

## Competing interests

EMA is an employee of Thermo Fisher Scientific, Inc.

## Authors’ contributions

RY, JEL and ZZ designed the experiments. ZZ performed initial characterizations of the miRNA-Seq biases and initial optimizations, prepared libraries from biological samples, and performed the primer extension assay and all bioinformatics analyses. JEL optimized 5′ and 3′ ligation reactions, prepared the optimized synthetic and HeLa libraries and performed the luciferase assays. KR performed the northern blotting. EMA provided the synthetic miRNAs. RY, JEL and ZZ wrote the manuscript. All authors read and approved the final manuscript.

## Supplementary Material

Additional file 1 **Tables S1 to S3 and figures S1 to S3. Table S1:** synthetic miRNA library. Complete list of 29 synthetic miRNAs and the primary sequences used in miRNA-Seq studies. **Table S2:** GC content of synthetic miRNA library. List of 29 synthetic miRNAs used in this study, their corresponding GC content listed as a percentage of total nucleotide number, and their observed cloning frequency listed as a percentage of total mapped miRNA reads. **Table S3:** oligonucleotides used in this study. List of oligonucleotides used in the ligation optimization and miRNA-Seq experiments. **Figure S1:** clustered miRNAs 143/145 exhibit differential quantification in a method-specific manner. (A) miRNA-Seq from mouse hair follicle. (B) qRT-PCR quantification of mouse epidermis determined by ΔΔCt method where sno25 serves as the reference gene. **Figure S2:** DNA oligos can be readily adenylated by MTH (Methanobacterium thermoautotrophicum) RNA ligase. 18% urea-PAGE of 3′ DNA linkers shows shift in electrophoretic mobility corresponding to 5′ adenylation where plus and minus signs indicate the presence or lack of MTH enzyme, respectively. **Figure S3:** enhanced miRNA-Seq approach is highly correlative for varying amounts of input RNA. Differing amounts of the 29 synthetic miRNA mix were subjected to enhanced miRNA-Seq. Click here for file
