# Supplementary material for: High-efficiency RNA cloning enables accurate quantification of miRNA expression by deep sequencing
Source: Genome Biol. 2013 Oct 7;14(10):R109. doi: 10.1186/gb-2013-14-10-r109 (PMC3983620; doi:10.1186/gb-2013-14-10-r109)
Supplement: Additional file 1 — Tables S1 to S3 and figures S1 to S3. Table S1: synthetic miRNA library. Complete list of 29 synthetic miRNAs and the primary sequences used in miRNA-Seq studies. Table S2: GC content of synthetic miRNA library. List of 29 synthetic miRNAs used in this study, their corresponding GC content listed as a percentage of total nucleotide number, and their observed cloning frequency listed as a percentage of total mapped miRNA reads. Table S3: oligonucleotides used in this study. List of oligonucleotides used in the ligation optimization and miRNA-Seq experiments. Figure S1: clustered miRNAs 143/145 exhibit differential quantification in a method-specific manner. (A) miRNA-Seq from mouse hair follicle. (B) qRT-PCR quantification of mouse epidermis determined by ΔΔCt method where sno25 serves as the reference gene. Figure S2: DNA oligos can be readily adenylated by MTH (Methanobacterium thermoautotrophicum) RNA ligase. 18% urea-PAGE of 3′ DNA linkers shows shift in electrophoretic mobility corresponding to 5′ adenylation where plus and minus signs indicate the presence or lack of MTH enzyme, respectively. Figure S3: enhanced miRNA-Seq approach is highly correlative for varying amounts of input RNA. Differing amounts of the 29 synthetic miRNA mix were subjected to enhanced miRNA-Seq. [file gb-2013-14-10-r109-S1.pdf]

Supplemental Figure S1. Zhang & Lee et al.

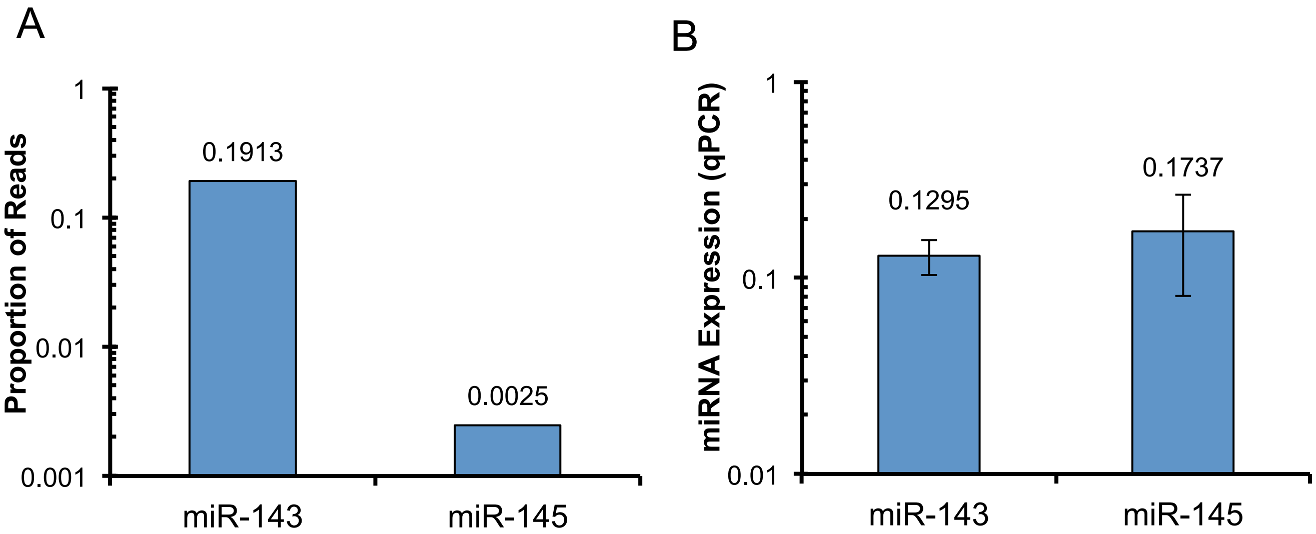

Supplemental Figure S1. Clustered miRNAs 143/145 exhibit differential quantification in a method-specific manner. (A) miRNA-seq from mouse hair follicle. (B) qRT-PCR quantification of mouse epidermis determined by $\Delta\Delta C_t$ method where sno25 serves as the reference gene.

Supplemental Figure S2. Zhang & Lee et al.

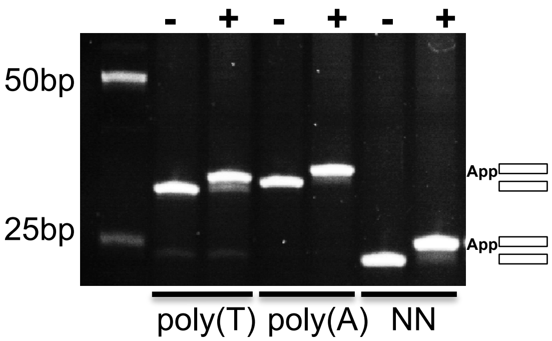

Supplemental Figure S2. DNA oligos can be readily adenylated by MTH. 18% Urea-PAGE of 3’ DNA linkers show shift in electrophoretic mobility corresponding to 5’ adenylation where + or – indicates presence or lack of MTH enzyme.

Supplemental Figure S3. Zhang & Lee et al.

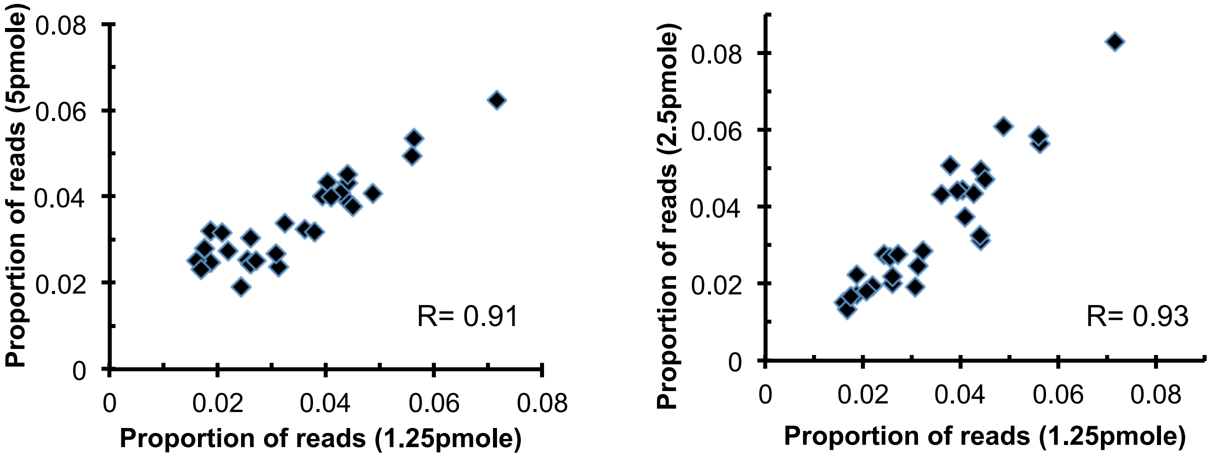

Supplemental Figure S3. Enhanced miRNA-seq approach is highly correlative for varying amounts of input RNA. Differing amounts of the 29 synthetic miRNA mix was subjected to Enhanced miRNA-seq.

Supplemental Table S1. Zhang & Lee et al.

Name	Sequence (listed 5' to 3')
let-7a	UGAGGUAGUAGGUUGUAUAGUU
let-7c	UGAGGUAGUAGGUUGUAUGGUU
let-7d	AGAGGUAGUAGGUUGCAUAGUU
let-7e	UGAGGUAGGAGGUUGUAUAGUU
let-7f	UGAGGUAGUAGAUUGUAUAGUU
let-7g	UGAGGUAGUAGUUUGUACAGUU
let-7i	UGAGGUAGUAGUUUGUGCUGUU
miR-103	AGCAGCAUUGUACAGGGCUAUGA
miR-125b-5p	UCCCUGAGACCCUAACUUGUGA
miR-141	UAACACUGUCUGGUAAAGAUGG
miR-143	UGAGAUGAAGCACUGUAGCUCU
miR-145	GUCCAGUUUUCCCAGGAAUCCCU
miR-182	UUUGGCAAUGGUAGAACUCACAC
miR-183	UAUGGCACUGGUAGAAUUCACU
miR-199a-5p	CCCAGUGUUCAGACUACCUGUUC
miR-19a	UGUGCAAAUCUAUGCAAAACUGA
miR-19b	UGUGCAAAUCCAUGCAAAACUGA
miR-200a	UAACACUGUCUGGUAACGAUGUU
miR-200b	UAAUACUGCCUGGUAAUGAUGA
miR-200c	UAAUACUGCCGGGUAAUGAUGGA
miR-203	GUGAAAUGUUUAGGACCACUAG
miR-203iso	UGAAAUGUUUAGGACCACUAG
miR-205	UCCUUCAUUCCACCGGAGUCUG
miR-214	ACAGCAGGCACAGACAGGCAGUA
miR-26a	UUCAAGUAAUCCAGGAUAGGCU
miR-26b	UUCAAGUAAUUCAGGAUAGGUU
miR-31	AGGCAAGAUGCUGGCAUAGCUG
miR-34a	UGGCAGUGUCUUAGCUGGUUGU
miR-429	UAAUACUGUCUGGUAAUGCCGU

Complete list of 29 synthetic miRNAs and the primary sequences used in miRNA-Seq. studies.

Supplemental Table S2. Zhang & Lee et al.

miRNA	GC Content (GC≥50% in red)	Cloning Frequency
mmu-miR-214	59.09%	0.0100%
mmu-miR-205	54.55%	0.0178%
mmu-miR-199a-5p	52.17%	0.0362%
mmu-miR-31	54.55%	0.0501%
mmu-miR-145	52.17%	0.0574%
mmu-miR-203	40.91%	0.2553%
mmu-miR-378	52.38%	0.3167%
mmu-miR-103	47.83%	0.3896%
mmu-miR-141	40.91%	0.5021%
mmu-miR-203iso	38.10%	0.6062%
mmu-miR-19a	34.78%	0.6583%
mmu-miR-34a	50.00%	0.7510%
mmu-miR-200c	43.48%	0.8411%
mmu-let-7g	36.36%	0.8661%
mmu-miR-21	36.36%	0.8735%
mmu-miR-26b	33.33%	1.0124%
mmu-miR-429	40.91%	1.1486%
mmu-let-7d	40.91%	1.6561%
mmu-miR-19b	39.13%	1.8493%
mmu-let-7i	40.91%	2.5455%
mmu-miR-200a	40.91%	3.4593%
mmu-miR-125b-5p	50.00%	5.8183%
mmu-miR-200b	36.36%	8.5892%
mmu-let-7f	31.82%	8.7352%
mmu-let-7e	40.91%	9.1801%
mmu-let-7c	40.91%	9.9007%
mmu-let-7a	36.36%	11.4990%
mmu-miR-143	47.62%	14.0575%
mmu-miR-26a	40.91%	14.3174%

List of 29 synthetic miRNAs used in this study, their corresponding GC content listed as a percent of total nucleotide number, and their observed cloning frequency listed as a percentage of total mapped miRNA reads.

Supplemental Table S3. Zhang & Lee et al.

Name	Molecule	Base Composition	Length	Step Used
poly(T)	DNA	5'phos-TTTTTTTTTTTCGTATGCCGTCTTCTGCTTG	31	3' Ligation
poly(A)	DNA	5'phos-AAAAAAAAAATCGTATGCCGTCTTCTGCTTG	31	3' Ligation
NN	DNA	5'phos-NNTCGTATGCCGTCTTCTGCTTG	23	3' Ligation
IDT L3	DNA	App-TTTAACCGCGAATTCCAG-ddC	19	3' Ligation
5' Adapter	RNA	GUUCAGAGUUCUACAGUCCGACGAUC	26	5' Ligation
5' Splint (+4)	DNA	NNNNGATCGTCGGACTGTAGAACTCTGAAC	30	5' Ligation
5' Adapter-NN	RNA	GUUCAGAGUUCUACAGUCCGACGAUCNN	28	5' Ligation

List of oligonucleotides used in the miRNA-Seq. protocol.
